# Evaluation of the diagnostic accuracy of Xpert® Mpox and STANDARD™ M10 MPX/OPX for the detection of monkeypox virus

**DOI:** 10.1016/j.jinf.2025.106413

**Published:** 2025-02

**Authors:** Alessandra Romero-Ramirez, Anushri Somasundaran, Konstantina Kontogianni, Jacob Parkes, Yusra Hussain, Susan Gould, Christopher T. Williams, Dominic Wooding, Richard Body, Hayley E. Hardwick, J. Kenneth Baillie, Jake Dunning, Malcolm G. Semple, CONDOR steering group, ISARIC 4C Investigators, Tom E. Fletcher, Thomas Edwards, Devy Emperador, Ana I. Cubas-Atienzar

**Affiliations:** a Centre for Drugs and Diagnostics, Liverpool School of Tropical Medicine, UK; b Department of Clinical Sciences, Liverpool School of Tropical Medicine, UK; c Royal Liverpool University Hospital NHS Foundation Trust, UK; d Manchester University NHS Foundation Trust, UK; e NIHR Health Protection Research Unit in Emerging and Zoonotic Infections, Department of Clinical Infection, Microbiology and Immunology, Institute of Infection, Veterinary and Ecological Sciences, University of Liverpool, Liverpool, UK; f Baillie Gifford Pandemic Science Hub, Institute for Regeneration and Repair, University of Edinburgh, UK; g Pandemic Sciences Institute, University of Oxford, UK; h Infectious Diseases Department, Royal Free London NHS Foundation Trust, London, UK; i Foundation of Innovative Diagnostics, Geneva, Switzerland

**Keywords:** Mpox, MPXV, Point-of-care (POC), Diagnostics, PCR, Monkeypox, Orthopoxvirus

## Abstract

**Objectives:**

Evaluation of diagnostic accuracy of two point-of-care (POC) molecular diagnostic tests for the detection of monkeypox virus (MPXV): Xpert® Mpox (Cepheid, Inc., USA) and STANDARD™ M10 MPX/OPX (SD Biosensor, Inc., Korea).

**Methods:**

Diagnostic accuracy of both POC platforms was evaluated using 53 upper-respiratory swabs (URS) and 32 skin lesions swabs (SS) collected from mpox and COVID-19 patients in the UK against the Sansure (Sansure Biotech Inc.) and CDC reference qPCR tests. The analytical sensitivity of both platforms was assessed using a viral isolate from II, B.1 lineage.

**Results:**

The overall sensitivity and specificity of the Xpert® Mpox was 97.67% [95% CI 87.71–99.94%] and 88.57% [95% CI 73.26–96.80%] and 97.44% [95% CI 86.52–99.94%] and 74.42% [95% CI 58.83–86.48%] comparing the Sansure and CDC qPCR, respectively and for the M10 MPX/OPX was 87.80% [95% CI 73.80–95.92%] and 76.60% [95% CI 61.97–87.70%] and 94.29% [95% CI 80.84–99.30%] and 86.67% [95% CI 73.21–94.95%] with the Sansure and CDC qPCR.

**Conclusion:**

The Xpert® Mpox had good diagnostic accuracy for both sample types while the M10 MPX/OPX clinical accuracy was deficient with URS. Our data supports the use of URS during the first 3 days of symptoms onset for mpox diagnosis.

## Highlights

• The Xpert® Mpox detected monkeypox virus (MPXV) DNA in more samples than the other assays suggesting better sensitivity.
• Xpert® Mpox fulfilled the WHO performance requirements using upper-respiratory swabs (URS) and skin lesion swabs (SS).
• M10 MPX/OPX fulfilled WHO recommended performance requirements using SS only.
• MPXV DNA was reliably detectable in SS for all days after onset of symptoms with all assays except M10 MPX/OPX.
• The use of URS for mpox diagnosis is recommended for use up to 3 days after onset of symptoms.

## Introduction

The highly infectious monkeypox virus (MPXV) is a double-stranded DNA virus belonging to the *Orthopoxviridae* family, which includes vaccinia, cowpox, and variola viruses.^1^ Orthopoxviruses are large viruses with a size range from 140–450 nanometers and a genome that contains over 200 genes.^2^ MPXV was identified in 1958 in captive cynomolgus macaques (*Macaca fascicularis*) that were transported from Singapore to Denmark^3^ and in 1970, the first known case of MPXV infection in a human from the Democratic Republic of the Congo (DRC) was reported.^4^ The WHO recommended “mpox” as the preferred term for human disease caused by MPXV in November 2022.^5^

Clinical manifestations of mpox infection include a vesiculopustular rash resembling that of smallpox, fevers, lymphadenopathy and a rash may affect palms and soles. Skin lesions may commence at the site of initially inoculation or exposure e.g. the anogenital region after transmission during sexual contact or at the site of a needlestick injury or bite.^6^ According to the Centres for Disease Control and Prevention (CDC), the incubation period is up to 21 days following/after viral exposure and the rash appears 1–4 days after initial flu-like prodrome.^7^ To confirm a clinical diagnosis, the World Health Organisation (WHO) advises testing for mpox as soon as possible in people who fit the suspected case definition.^8^ Laboratory-based nucleic acid amplification tests (NAAT) are the primary method used for mpox diagnosis.^9^ Laboratory-based PCR testing requires specialist equipment, up front DNA extraction, and skilled personnel to perform such tests. Many cases in low- and middle-income countries (LMIC) remain unreported due to a lack of decentralised diagnostic resources in the area, and issues with the current healthcare system and civil upheaval.

In May 2022, an mpox outbreak spread to over 110 countries with over 86,000 confirmed cases.^10^ The number of infections during the 20th century has already been surpassed by cases after the 2022 outbreak.^11^ At that time, in the United Kingdom (UK), all clades of mpox were classified as a High Consequence Infectious Disease (HCID) and patients were looked after in specially designed HCID treatment facilities run by a nationwide network.^12^ From August 2018 to September 2021, 7 mpox cases were identified in the UK and received treatment in HCID centres (4 imported cases and 3 secondary cases).^12^ The discovery of the first mpox case of the global outbreak was on May 7, 2022, a person who travelled from Nigeria^13^ and as of June 8, 2022, there were 336 laboratory-confirmed cases in the UK. Most of these cases were identified in men [99%], who were primarily residents of London [81%].^14^ For the first time, community transmission was reported in the UK, which was mainly through intimate person-to-person contact, often involving sexual activity and mostly unrelated to travel from endemic countries.^15^

The increasing global cases of mpox following the 2022 outbreak brought to light the difficulties in meeting the increased and erratic demand of decentralised diagnostics for different virus prone to outbreaks. Another public health emergency of international concern (PHEIC) was declared by WHO on 14th August 2024 given the significant increase in mpox cases which has the potential to spread beyond Africa.^16^ This highlighted the urgent need for the rational development of rapid diagnostic methods for emerging pathogens such as for MPXV as a priority. As a result, several point-of-care (POC) NAAT platforms were developed to identify MPXV at the POC or for near-patient testing since the 2022 outbreak.^17^, ^18^ POC NAAT offer higher sensitivity and specificity compared to antigen-based POC tests and are equal to laboratory-quality testing without the requirement for sophisticated laboratory facilities,^19^ requiring less operational training and fewer sample preparation steps compared to lab-based PCR.

Prompt isolation and optimal clinical care are all dependent on an accurate diagnosis of MPXV infection. In this study, we evaluated the diagnostic accuracy of two new POC NAAT, Xpert® Mpox (Cepheid, Inc., Sunnyvale, CA, USA) and STANDARD™ M10 MPX/OPX (SD Biosensor, Inc., Suwon, Korea), for the detection of MPXV on skin lesion and upper-respiratory swabs.

## Methodology

### Study design

Skin lesion swabs (SS) (n=30) and upper-respiratory swab (URS) samples (n=23, [nasopharyngeal=1, oropharyngeal=22]) in universal transport media (UTM, RT-UTM Copan, Italy) from a cohort of 16 mpox patients enrolled at the Royal Liverpool University Hospital, Sheffield Teaching Hospital NHS Foundation Trust, and Royal London Hospital were used for this study. Patients were recruited during the last two outbreaks of mpox in the UK, 2018 and 2022. Patients were consented under the WHO ISARIC 4C Comprehensive Clinical Characterisation Collaboration Protocol for severe emerging infections [ISRCTN66726260],^20^ ethical approval was obtained from the National Research Ethics Service and the Health Research Authority (IRAS ID:126600, REC 13/SC/0149). All mpox patients were diagnosed by sending samples to the UK Health Security Agency (UKHSA) for testing using qPCR. In addition to the samples from mpox-positive patients, to fulfil with the minimum number of negative swab specimens for mpox diagnostic evaluations recommended by the by the FDA,^15^ a set of 32 leftover nasopharyngeal samples in UTM (RT-UTM Copan, Italy) from prior COVID-19 studies^21^, ^22^, ^23^, ^24^ were used as mpox negative controls. These were collected under the Facilitating AcceLerated Clinical validation Of Novel diagnostics for COVID-19^22^ and ethical approval was obtained from the National Research Ethics Service and the Health Research Authority (IRAS ID:28422, REC: 20/WA/0169). All samples were aliquots stored at −80 °C and thawed for the first time for this study. Samples were processed and tested at the Biosafety Level 3 (BSL3) Laboratories of the Liverpool School of Tropical Medicine (LSTM) as previously described.^19^

### MPXV PCR reference assays

The DNA was extracted from 200 µl of UTM using the QiAamp96 Virus Qiacube HT kit (Qiagen, Germany). Two reference PCR tests were used, the commercially available CE-IVD Sansure qPCR kit (Monkeypox virus Nucleic Acid Diagnostic Kit, Sansure Biotech Inc.), and the CDC Monkeypox virus Generic Real-Time PCR Test.^25^ Both lab-based PCR tests were used as reference tests as the CDC qPCR is widely used, the Sansure qPCR kit is CE-IVD marked and both have successfully demonstrated to detect MPXV clades I, IIa and IIb.^26^, ^27^ The PCRs were performed on the QuantStudio 5 (ThermoFisher, USA) following the manufacturer instructions (Sansure Biotech Inc.) and the CDC guidelines.^25^ The CDC qPCR was performed using the QuantiFast Pathogen PCR kit (Qiagen, Germany).

### MPXV POC index NAAT

Two rapid molecular POC platforms which perform automated sample processing and qPCR to detect viral DNA were evaluated in the study: Xpert® Mpox and the STANDARD™ M10 MPX/OPX (M10 MPX/OPX hereinafter). The platforms were selected following an expression of interest launched by FIND (www.finddx.org) and a scoring process based on defined criteria. The evaluation of the platforms at LSTM was done in BSL3 laboratories.

The Xpert® Mpox assay is authorised for use under FDA Emergency Use Authorization (EUA) and provides semiquantitative detection and differentiation between MPXV clade II (two undisclosed targets) and non-variola *Orthopoxvirus* (target OPXV-E9L NVAR gene) DNA, respectively.^28^ A Sample Processing Control (SPC), a Sample Adequacy Control (SAC), and a Probe Check Control (PCC, not included in the algorithm, used as quality control) are also included in the cartridge utilised by the GeneXpert® instrument.^29^ The LOD reported by the manufacturer is 2.12 × 10^1^ genome copies/mL for MPXV with an approximate time of 36 min to get the results.^30^ The tests were performed according to the manufacturer’s instructions. Briefly, 300 µl sample were transferred to the sample chamber of the Xpert® Mpox test cartridge and loaded onto the GeneXpert® Instrument System platform. The results were automatically interpreted by the GeneXpert® System based on the Ct values results. A sample was called positive when it was positive for the 2 MPXV targets (OPXV, SAC, SPC could be either positive or negative); negative result when it was negative for MPXV and OPXV but positive for SAC and SPC; a positive result for non-variola OPXV when it was positive for the OPXV target, negative for MPXV (SAC and SPC can be either positive or negative); and invalid when it was negative for both viral targets and controls or when only one control was positive but both viral targets negative.

The M10 MPX/OPX assay is for Research Use Only and provides semiquantitative detection and differentiation between MPXV and OPXV DNA using E9L and G2R gene targets, respectively. The LOD as reported by the manufacturer is 1.0 ×10^2^ genome copies/mL and can be used on different specimen types such as skin lesion material, whole blood, oropharyngeal swabs and plasma.^31^ The tests were performed according to the manufacturer’s instructions. Briefly, 300 µl of sample were transferred to the sample chamber of the cartridge and loaded onto the STANDARD™ M10 platform. After 1 h, the results with the corresponding Ct values were displayed on the STANDARD™ M10 screen and the results were automatically interpreted. A sample was considered positive for MPXV when MPXV and OPVX targets were positive (IC can be either positive or negative), positive result for OPXV when MPXV was negative and OPXV was positive (IC can be either positive or negative), negative when only the IC was positive and invalid when all targets were negative or when only the MPXV target was positive. During the COVID-19 pandemic, SD Biosensor developed the M10 SARS-CoV-2, a molecular in vitro diagnostic assay able to detect SARS-CoV-2 viral RNA that also uses the M10 platform as the MPX/OPX assay^32^ with 100% sensitivity and 100% specificity.^33^, ^34^

### Analytical limit of detection of qPCR reference assays and POC index NAATs

An MPXV strain (Slovenia_MPXV-1_2022, isolate 2225/22 Slovenia ex Gran Canaria) from the lineage II, B.1 (European Virus Archive Global EVAg, Marseille, France) was cultured in Vero C1008 (ECACC 85020206) (Vero E6 cells) obtained from the European collection of authenticated cell cultures (ECACC) in Dulbecco’s Modified Eagle Medium (DMEM, Gibco, USA) plus 10% foetal bovine serum (FBS, Gibco, USA) and 1% Penicillin/Streptomycin solution (Gibco, USA) to generate the MPXV stock. Frozen aliquots of the fourth passage of the virus were quantified via plaque assay. The MPXV stock was used to investigate the limit of detection (LOD) of both Xpert® Mpox and M10 MPX/OPX assays. A fresh aliquot was serially diluted from 1.0×10^4^ plaque forming units (pfu)/mL to 1.0 ×10^2^ pfu/mL using UTM media. Each dilution was tested in triplicate and UTM was used as negative control following previous work.^21^, ^35^ The LOD was defined as the lowest dilution where all three replicates were positive. DNA from the serial dilutions was extracted using the QiAamp96 Virus Qiacube HT kit and viral copy numbers per mL (copies/mL) were calculated using a standard curve of quantified synthetic DNA (G2R gene) in the QuantStudio 5 tested using the CDC PCR. Synthetic DNA (Eurofins Genomics, UK) was re-hydrated in Tris-EDTA buffer and concentration was quantified using Qubit™ SSDNA Quantification Assay kit (ThermoFisher, USA). Standard curve was prepared using an eight 10-fold serial dilution series with 5 replicates per dilution.

### Statistical analysis

The sensitivity and specificity of the index tests were calculated with 95% confidence interval in comparison to both reference PCR assays, including stratification by cycle threshold (Ct) value. Prior to the analysis, a normality test was performed using the Shapiro-Wilk test (p<0.05). Differences between the Ct values (expressed as mean± standard deviation [SD]) in sample groups were assessed using the paired Student’s t-test. Differences in the frequency of MPXV detection by sampling date were analysed using Chi-squared test and Fisher's exact test. Statistical significance was set for a p <0.05. The statistical analysis was performed using GraphPad Prism version 8.0.0, GraphPad Software (Boston, USA).

## Results

### Clinical evaluation

The demographic and clinical characteristics of the participants are shown in Table 1. The 16 positive patients with mpox were assessed at the three hospitals during the study period. All individuals were men (100%) with a mean age of 35.1 years (range 24–58 years). The median days from onset of symptoms was 8 with the most common symptoms being skin lesions (100%), skin rashes (87.5%) and fever (68,75%). The results shown in Table 1 included only the mpox-positive patients (n=16). The negative cohort (COVID-19 patients) was not included in Table 1 as these were from a population not suspected from MPXV infection.

**Table 1:** Clinical characteristics of mpox patients from UK used for the evaluation of both molecular platforms.

**Characteristic**	
Age [mean (min-max), N]	35.1 (24–58), 16
Gender [%M, (n/N)]	100%; (16/16)
Days from symptom onset [median (Q1-Q3); N]	8 (4.25 - 12.75); 16
Days < 0–3 (n, %)	1, 6.25%
Days 4–7 (n, %)	6, 37.5%
Days 8+ (n, %)	9, 56,25%
**Symptoms** [% (n/N)]	
Skin lesions	100% (16/16)
Skin rashes	87.5% (14/16)
Fever	68.75% (11/16)
Flu like symptoms	25% (4/16)
Headache	25% (4/16)
Sore throat	25% (4/16)
Cough	6.25% (1/16)
Diarrhoea	6.25% (1/16)
Chest pain	0% (0/16)
Abdominal pain	0% (0/16)
Nausea	0% (0/16)
Vomiting	0% (0/16)
Painful Urination	0% (0/16)

Fourteen of the 23 URS and 25 of 30 SS collected from mpox positive patients were positive by the CDC qPCR. When using the Sansure qPCR, 1 further URS (4.3%) and 4 SS (13%) were positive. The mean Ct value when using the Sansure and CDC qPCR were 30.09 (**±** 5.70) and 27.54 (**±**5.87), respectively. There was no significant difference between the mean difference in Ct values when compared CDC and Sansure qPCR for any of the sample types (p-value= 0.34). As expected, all 32 UTM samples collected from the COVID-19 cohort were negative for MPXV using both reference qPCR tests.

The overall clinical sensitivity for the Xpert® Mpox assay using both sample types was 97.67% [95% CI 87.71 – 99.94%] with the Sansure qPCR and 97.44% [95% CI 86.52 – 99.94%] with the CDC qPCR. In addition, the clinical specificity for both sample types was 88.57% [95% CI 73.26 – 96.80%] when tested with the Sansure qPCR and 74.42% [95% CI 58.83 – 86.48%] comparing to the CDC qPCR. The Ct values by sample type are found in Table 2, Table 3. The overall percentage of agreement was 90.3% [95% CI 81.7– 95.7%] and 91.5% [95% CI 83.2– 96.5%], when using the Sansure qPCR and CDC qPCR, respectively.

**Table 2:** Results and clinical sensitivity and specificity of the Xpert® Mpox assay and M10 MPX/OPX using URS with valid results from mpox (n=23) and COVID-19 patients (n=32).

	Results	Sansure qPCR			CDC qPCR		
		Positive	Negative	Total	Positive	Negative	Total
**Xpert**® **Mpox**	Positive	15	6	21	14	7	21
	Negative	0	31	31	0	31	31
	Total^a^	15	37	52	14	38	52
	Clinical sensitivity (95% CI)	100% (78.20–100%)			100% (76.24–100%)		
	Ct <25 [95% CI, N]	100% (15.81–100%), 2			100% (39.76–100%), 4		
	Ct <35 [95% CI, N]	100% (69.15–100%), 10			100% (69.15–100%), 10		
	Ct <40 [95% CI, N]	100% (78.20–100%), 15			100% (76.84–100%), 14		
	Clinical specificity (95% CI)	83.78% (67.99–93.81%)			81.58% (65.67–92.26%)		
**M10 MPX/OPX**	Positive	10	2	12	10	2	12
	Negative	4	36	40	2	38	40
	Total^a^	14	38	52	12	40	52
	Clinical sensitivity (95% CI)	71.43% (41.90–91.61%)			71.43% (41.90–91.61%)		
	Ct <25 [95% CI, N]	100% (15.81–100%), 2			100% (15.81–100%), 2		
	Ct <35 [95% CI, N]	100% (66.37–100%), 9			100% (66.37–100%), 9		
	Ct <40 [95% CI, N]	71.43% (41.90–91.61%), 10			100% (69.15–100%), 10		
	Clinical specificity (95% CI)	92.11% (78.62–98.34%)			94.59% (81.81–99.34%)		

a Of the 55 URS, 3 were invalid with Xpert® Mpox and M10 MPX/OPX.

**Table 3:** Results and clinical sensitivity and specificity of the Xpert® Mpox assay and M10 MPX/OPX assays using skin lesion swabs (SS) with valid results (n=30).

	Results	Sansure qPCR			CDC qPCR		
		Positive	Negative	Total	Positive	Negative	Total
**Xpert**® **Mpox**	Positive	27	2	29	24	4	28
	Negative	1	0	1	1	1	2
	Total	28	2	30	25	5	30
	Clinical sensitivity (95% CI)	96.43% (81.65–99.91%)			96% (79.65–99.90%)		
	Ct <25 [95% CI, N]	100% (63.06–100%), 8			100% (73.54–100%), 12		
	Ct <33 [95% CI, N]	100% (63.03–100%), 17			100% (83.16–100%), 20		
	Ct <40 [95% CI, N]	96.43% (81.65–99.91%), 30			96% (79.65–99.90%), 30		
	Clinical specificity (95% CI)^a^	NA			NA		
**M10 MPX/OPX**	Positive	26	1	27	23	4	27
	Negative	1	0	1	0	1	1
	Total^b^	27	1	28	23	5	28
	Clinical sensitivity (95% CI)	96.30% (81.03–99.91%)			100% (85.18–100%)		
	Ct <25 [95% CI, N]	100% (69.15–100%), 10			100% (73.54–100%), 12		
	Ct <33 [95% CI, N]	100% (79.41–100%), 16			100% (82.35–100%),19		
	Ct <40 [95% CI, N]	96.30% (81.03–99.91%), 28			100% (85.18–100%), 23		
	Clinical specificity (95% CI)^a^	NA			NA		

a Only one SS was negative using the CDC qPCR so specificity was not calculated for this sample type.

b Of the 30 total samples, 2 SS were invalid with M10 MPX/OPX assay.

Three URS were invalid with Xpert® Mpox assay (5.45%, 3/55; 2 URS from mpox patients and 1 COVID-19 patient) while all of SS were valid. (Table 2, Table 3). The specificity was 83.78% [95% 78.20 - 100%] and 81.58% [95% CI 65.67 – 92.26%] for URS using Sansure and CDC PCR. Specificity could not be accurately calculated for SS due to the lack of negative specimens using the reference tests.

The overall sensitivity for the M10 MPX/OPX using both sample groups were 87.80% [95% CI 73.80 – 95.92%] with the Sansure qPCR and 94.29% [95% CI 80.84 – 99.30%] with the CDC qPCR. Moreover, the clinical specificity for both sample types was 76.60% [95% CI 61.97 – 87.70%] with Sansure and 86.67% [95% CI 73.21 – 94.95%] with the CDC qPCR. The specificity was 92.11% (78.62–98.34%) and 94.59% [95% CI 81.81 - 99.34%] using URS compared to Sansure and CDC PCR, respectively. The overall percentage of agreement was 91.3% [95% CI 82.8 – 96.4%] and 95.0% [95% CI 87.7 – 98.6%] with the Sansure qPCR and CDC qPCR, respectively. All SS were positive with both reference assays except for 1 sample using the CDC PCR, therefore specificity could not be accurately calculated for this sample type. Three URS (5.45%, 3/55 all mpox patients) and 2 SS (6.66%, 2/30) were invalid using the M10 MPX/OPX.

**Fig. 1:**
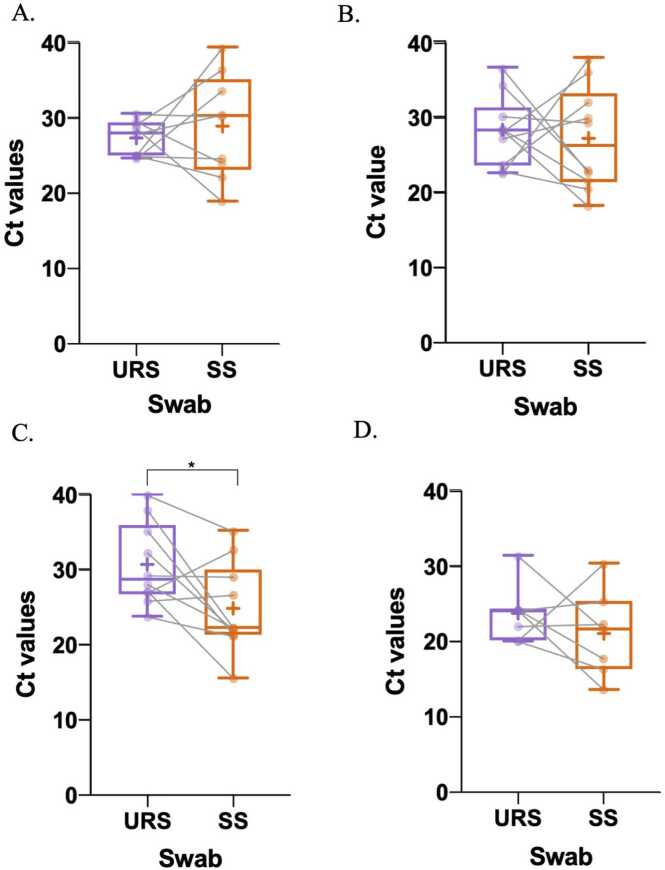
Boxplot of the Ct values from paired URS and SS tested by A. Sansure qPCR (n=9), B. CDC qPCR (n=10), C. Xpert® Mpox (n=10) and D. M10 MPX/OPX (n=7). The whiskers show the maximum and minimum values and the vertical line the median. There was a significant difference (p-value <0.05) between paired URS and SS when evaluated with the Xpert® Mpox assays with higher Ct values in the URS group.

The Ct values of paired samples were also compared and evaluated for each qPCR reference assay and POC index NAAT (Fig. 1A-1D). Nine, 10, 10 and 7 URS and SS paired samples were positive for Sansure qPCR, CDC, Xpert Mpox and M10 MPX/OPX. No significant differences in Ct values were found between URS and SS sample groups when using the Sansure qPCR, CDC and M10 MPX/OPX (p-value=0.54, 0.73 and 0.37, respectively). The analysis of the paired samples using the Xpert® Mpox assay showed higher Ct values in the URS group compared to the SS group (p-value=0.03) with mean Ct values of 30.58 (± 5.48) and 24.75 (±5.98) for URS and SS.

Overall, the higher positivity rates for detecting MPXV DNA in clinical samples from mpox patients were reported by the Xpert® Mpox (n=50/53), followed by Sansure qPCR (n=44/53), CDC qPCR (n=41/53) and M10 MPX/OPX (n=37/53) and this difference was statistically significant for URS (p= 0.015) but not for SS (p= 0.692).

**Fig. 2:**
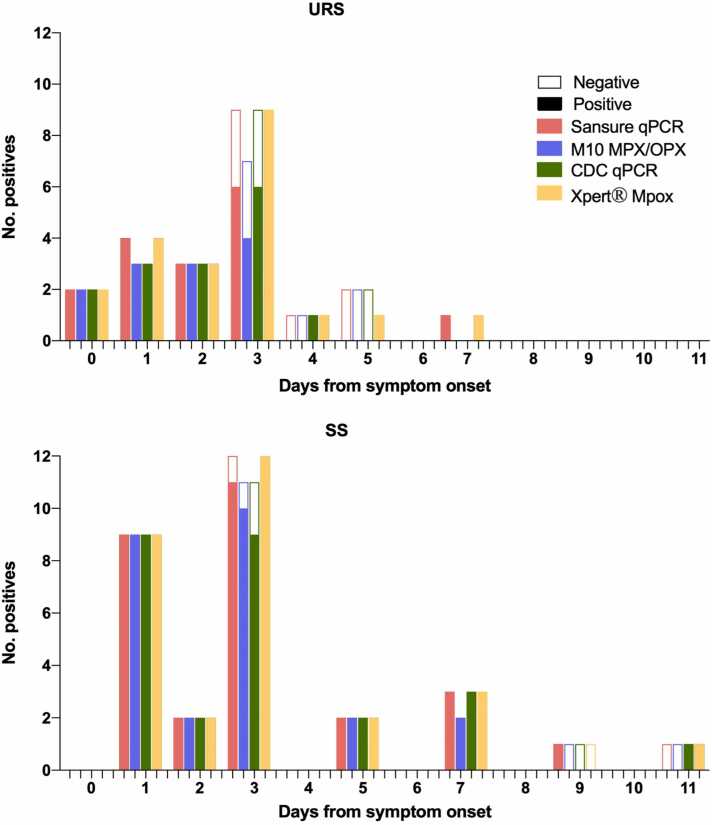
Number of positive samples according to sampling date from symptoms onset for SS and URS. The Ct values as a proxy for viral loads were analysed by sampling day from onset of symptoms and higher Ct values were observed as the sampling day increased in URS for Sansure qPCR (p = 0.0093, r=0.58 95% 0.12–0.84) CDC qPCR (p = 0.0444, r=0.45 95% −0.08–0.8) and Xpert® Mpox (p = 0.0024, r=0.59 95% 0.21–0.81) but not for M10 (p = 0.1752, r=0.30 95% −0.33–0.74). No correlation was observed between viral loads and sampling date in SS (Fig. 3).

The number of MPXV-positive samples depending on the sampling collection day from the onset of symptoms was evaluated for all samples and all PCR tests (Fig. 2). URS collected from MPXV patients more than 3 days after the symptom onset were less likely to have detectable levels of virus using all PCR assays used in the study (Sansure p = 0.017, CDC p = 0.033, Xpert® Mpox p = 0.04 and M10 MPX/OPX p = 0.014). URS collected from MPXV patients more than 2 days after the symptom onset were less likely to present the virus to detectable levels by Sansure (p = 0.014) and M10 MPX/OPX (p = 0.007). This was not significant for the SS for any collection date (all p values > 0.05) except for M10 MPX/OPX among SS collected more than 5 days after onset of symptoms (p = 0.022).

**Fig. 3:**
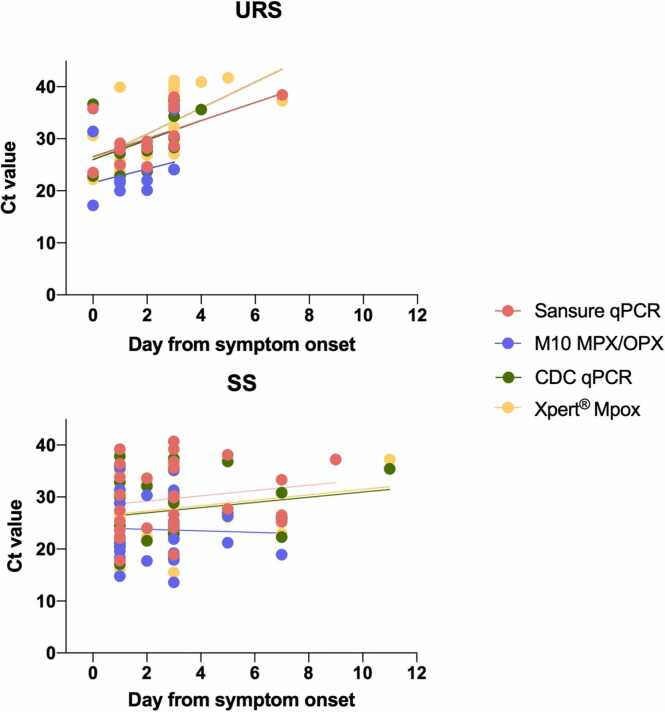
Plot of the Ct values of the four platforms by collection day from symptom onset using SS (n=30) and URS (n=23) from MPXV positive patients. Data points are individual clinical samples, with SS sampling from different lesions.

## Analytical evaluation

**Fig. 4:**
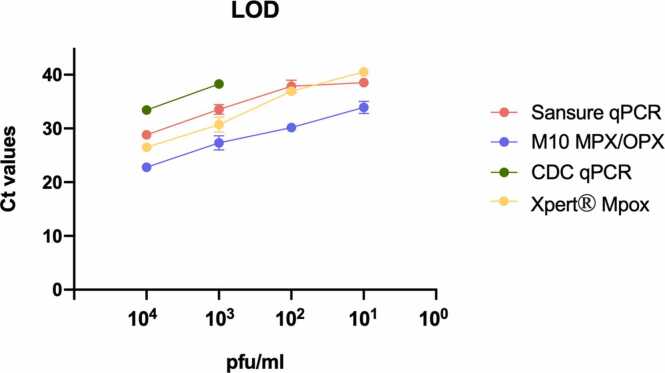
Relationship between Ct values and viral load using both qPCR reference assays and POC index NAATs.

The LOD was 1.0×10^1^ pfu/mL for Xpert® Mpox and M10 MPX/OPX, 1.0×10^2^ pfu/mL for Sansure qPCR and 1.0×10^3^ pfu/mL for the CDC qPCR (Fig. 4). The approximated viral copy number of the LOD was calculated for all the assays and was at ≈1.31×10^2^ copies/mL for Xpert® Mpox and M10 MPX/OPX, ≈1.3×10^3^ copies/mL for Sansure qPCR and ≈1.3×10^4^ copies/mL for CDC qPCR.

## Discussion

The primary aim of this study was to evaluate the diagnostic performance of two POC NAAT, Xpert® Mpox and M10 MPX/OPX. Rapid molecular diagnostic tests offer several advantages to laboratory-based PCR methods such as minimal sample processing, automated results readout and rapid availability of results to speed up clinical decision-making for timely management in outbreak situations, hence it is critical to assess their diagnostic accuracy.

The Xpert® Mpox test is designed to be used with lesion swabs.^36^ Previous studies have evaluated the accuracy for detection of MPXV in crusts and vesicular swabs samples in DRC showing a sensitivity of 98% and a specificity of 100% in both sample types^37^ and in oropharyngeal, lesions and anal swabs in Georgia, USA, with a sensitivity of 100% and specificity of 83.3% to 90.9% depending on sample type.^38^ The published data aligns with our results when using the platform with both lesion and upper-respiratory swabs. In this study, the Xpert® Mpox detected MPXV DNA from clinical samples that were negative by the reference lab-based qPCR Sansure and CDC, suggesting greater sensitivity. The greater sensitivity of the Xpert® Mpox compared to a reference lab-based MPXV PCR has also been observed elsewhere.^38^ This could be due to the larger sample volume used in the Xpert® Mpox (300 µl) compared to the volume used in the lab-based qPCR tests (2.5 µl and 10 µl of 50 µl eluted DNA extracted from 200 µl of UTM for CDC and Sansure respectively). The GeneXpert platform has been widely used for the detection of several infectious diseases, including SARS-CoV-2, *Mycobacterium tuberculosis* with rifampicin resistance, methicillin-resistant *Staphylococcus aureus*, and Ebola virus disease also showing high sensitivity and specificity.^39^, ^40^, ^41^ However, the price of the Xpert® Mpox cartridges is currently a limitation for deployment in LMICs (currently **≈** $20 USD). There is an urgent call for a price drop to $5 USD in response to the current PHEIC (ref). An advantage of the GeneXpert platform is that it is the same used for the Xpert MTB/RIF for TB testing and is widely available in TB endemic countries.

The M10 MPX/OPX can be used on different specimen types such as skin lesion material, whole blood, oropharyngeal swabs and plasma.^31^ Compared to Xpert® Mpox, sensitivity was lower despite of manufacturer claims of having the same analytical LOD (100 copies/mL) as the Sansure reference test. The M10 MPX/OPX assay is for research use and skin lesion material, whole blood, oropharyngeal swabs and plasm. In this study, the M10 MPX/OPX platform detected less MPXV positive samples than the other tests, suggesting lower sensitivity. A previous study evaluating the diagnostic accuracy of the M10 MPX/OPX test, showed lower sensitivity compared to the lab-based qPCR RealStar® OPX-1,^42^ aligning with the results obtained in the present study.

The WHO recommends the use of skin lesions for laboratory confirmation of MPXV infection whenever possible.^9^ Our study indicates that URS can be used as a reliable alternative sample type to SS for patients sampled within the first 3 days of symptoms onset. This presents an advantage as the use of URS for POC testing in suspected cases can be used to diagnose mpox in patients without typical skin lesions including those who may be in the prodromal phase of the disease when skin lesions have not appeared yet. The use of URS for mpox diagnosis early in the disease can be particularly beneficial for monitoring contacts of positive cases for rapid detection, isolation and patient management. However, the use of SS for MPXV detection was more robust among samples collected from patients regardless of the time from symptom onset, except for M10 MPX/OPX that showed poor sensitivity in skin lesion swabs collected from patients more than 5 days after symptom onset. This provides key information for choosing the adequate sample type and tests, specifically for when patients present to the clinic several days after the disease onset. The Ct value of paired URS and SS showed no significant differences except for the Xpert® Mpox platform. These results differ from previous studies where they observed that lesion samples presented a viral load 3 orders of magnitude higher than URS^43^ and suggest that URS testing offers no additional information for the diagnosis in individuals presenting skin lesions.^43^, ^44^, ^45^ The lower sensitivity obtained in URS among these collected from patients more than 3 days after symptom onset can be attributed to viral clearance occurring earlier in the oropharynx sample than in skin lesions.^46^

Based on the target products profile (TPP) for tests used for mpox diagnosis within health care settings and laboratories published by the WHO, the minimal and optimal clinical sensitivity should be ≥ 95% and ≥ 97%, and minimal and optimal clinical specificity should be ≥ 97% compared to a reference molecular method.^47^ The results obtained using the Xpert® Mpox assay met the minimal clinical sensitivity using SS and optimal sensitivity using URS regardless of the qPCR used as reference method. In the case of the MPX/OPX assay, the minimal and optimal clinical sensitivity was met with SS when compared to Sansure and CDC qPCR reference tests; however, the sensitivity using URS did not fulfil the minimum clinical sensitivity regardless of the qPCR reference assay used. False positive results in the index tests have been attributed to lower sensitivity of the reference test compared to the index tests since all the “false positive” results were obtained from MPXV positive patients and both Xpert® Mpox and M10 MPX/OPX had a lower LOD than the reference tests used in this study. This is of importance as reference lab-based qPCR tests such as the widely used CDC protocol may fail to diagnose true MPXV-positive samples, and a composite reference standard should be determined.

The WHO recommends using laboratory-based nucleic acid amplification testing (NAAT) to confirm an MPXV infection.^9^ The primers and probes used in the current MPXV generic qPCR test created by the CDC^25^ differ significantly due to genetic variations in >1000 available sequenced MPXV genomes impacting on the sensitivity and specificity of the test.^48^ This could be a possible explanation of the higher LOD and, consequently, lesser number of positive samples detected with the CDC qPCR compared with Sansure qPCR.

In this study we used frozen samples due to low prevalence causing difficulties for fresh samples and prospective evaluation. However, the IFU of both index tests indicate they can detect MPXV in frozen samples as well as samples stored at 4 °C and room temperature.^36^ The lack of negative skin lesion swab specimens is a limitation of the study as we could not calculate specificity using this sample type.

In conclusion, the Xpert® Mpox demonstrated the greatest diagnostic accuracy for POC testing and the use of URS as alternative sample type to skin lesions has been shown to perform well in samples collected within 3 days from onset symptoms. This study adds important insights on the diagnostics of mpox, which are very much needed considering the 2024 PHEIC declaration and an ongoing need for accurate diagnostics that can be used in affected countries.

## Funding

This work was funded as part of FIND’s work as coconvener of the diagnostics pillar of the Pandemic Threats Programme. ISARIC 4C was funded from the 10.13039/501100000272National Institute for Health Research [award CO-CIN-01], the 10.13039/501100000265Medical Research Council [grant MC_PC_19059] and by Liverpool Pandemic Institute and the 10.13039/501100000272National Institute for Health Research Health Protection Research Unit (NIHR HPRU) in Emerging and Zoonotic Infections at University of Liverpool in partnership with UK Health Security Agency (UK-HSA), in collaboration with Liverpool School of Tropical Medicine and the University of Oxford [NIHR award 200907], 10.13039/100004440Wellcome Trust and 10.13039/501100002992Department for International Development [215091/Z/18/Z], and the 10.13039/100000865Bill and Melinda Gates Foundation [OPP1209135], and Liverpool Experimental Cancer Medicine Centre for providing infrastructure support for this research (Grant Reference: C18616/A25153).

The FALCON study was funded by the National Institute for Health Research, Asthma United Kingdom, and the 10.13039/501100000351British Lung Foundation. This work is partially funded by the National Institute for Health Research (NIHR) Health Protection Research Unit in Emerging and Zoonotic Infections (200907), a partnership between the United Kingdom Health Security Agency (UKHSA), The 10.13039/501100000836University of Liverpool, The 10.13039/501100000769University of Oxford, and 10.13039/100014976LSTM.

## Author contributions

The study was conceived and designed by AICA, JD and DE. Laboratory work was conducted by AS, JP, YH, DW, NK, CTW and ARR, with supervision by AICA and TE. Data collections were conducted by AS, JP, YH, HH, NK, and SG. Data analysis and interpretation were conducted by ARR and AICA. The initial manuscript was prepared by ARR and AICA. Funding was acquired by AICA, the CONDOR steering group and ISARIC 4C investigators. Oversight of participant recruitment was performed by AICA, the CONDOR steering group and ISARIC 4C investigators. All authors edited and approved the final manuscript.

## Declaration of Competing Interest

The authors declare that they have no known competing financial interests or personal relationships that could have appeared to influence the work reported in this paper.

## References

[1] Parker S, Schultz D, Meyer H, Buller R. Smallpox and Monkeypox Viruses; 2014. 10.2217/17460913.2.1.17. Accessed on 15 January 202

[2] Titanji B.K., Tegomoh B., Nematollahi S., Konomos M., Kulkarni P.A. (2022). Monkeypox: a contemporary review for healthcare professionals. Open Forum Infect Dis.

[3] Magnus P, Andersen EK, Petersen KB, Birch Andersen A. A pox-like disease in cynomolgus monkeys; 1959. 10.1111/j.1699-0463.1959.tb00328.x.

[4] Breman J.G., Steniowski M., Zanotto E., Gromyko A., Arita I. (1980). Human monkeypox, 1970-79. Bull World Health Organ.

[5] World Health Organization. WHO recommends new name for monkeypox disease; 2022 [updated 28 November 2022]. https://www.who.int/news/item/28-11-2022-who-recommends-new-name-for-monkeypox-disease. Accessed on 15 January 2025.

[6] McCollum A.M., Damon I.K. (2014). Human monkeypox. Clin Infect Dis.

[7] Centers for Disease Control and Prevention. Mpox: signs and symptoms; 2024. https://www.cdc.gov/mpox/signs-symptoms/index.html. Accessed on 15 January 2025.

[8] Organization W.H. (2022).

[9] Organization WH. Diagnostic testingfor the monkeypox virus (MPXV): interim guidance, 9 November 2023. Diagnostic testing for the monkeypox virus(MPXV): interim guidance, 9 November 2023; 2023. https://www.who.int/publications/i/item/WHO-MPX-Laboratory-2023. Accessed on 15 January 2025.

[10] Organization W.H. (2022).

[11] Pan D., Nazareth J., Sze S., Martin C.A., Decker J., Fletcher E. (2023). Transmission of monkeypox/mpox virus: a narrative review of environmental, viral, host, and population factors in relation to the 2022 international outbreak. J Med Virol.

[12] Adler H., Gould S., Hine P., Snell L.B., Wong W., Houlihan C.F. (2022). Clinical features and management of human monkeypox: a retrospective observational study in the UK. Lancet Infect Dis.

[13] Organization WH. Multi-country monkeypox outbreak: situation update; 2022. https://www.who.int/emergencies/disease-outbreak-news/item/2022-DON393. Accessed on 15 January 2025.

[14] Agency UHS. Investigation Into Monkeypox Outbreak in England. United Kingdom Health Security Agency; 2022. https://www.gov.uk/government/publications/monkeypox-outbreak-technical-briefings/investigation-into-monkeypox-outbreak-in-england-technical-briefing-1. Accessed on 15 January 2025.

[15] Girometti N., Byrne R., Bracchi M., Heskin J., McOwan A., Tittle V. (2022). Demographic and clinical characteristics of confirmed human monkeypox virus cases in individuals attending a sexual health centre in London, UK: an observational analysis. Lancet Infect Dis.

[16] Organization WH. WHO Director-General declares mpox outbreak a public health emergency of international concern; 2024. https://www.who.int/news/item/14-08-2024-who-director-general-declares-mpox-outbreak-a-public-health-emergency-of-international-concern. Accessed on 15 January 2025. PMC11376700 39218470

[17] Agency UFaDAF. Monkeypox (mpox) Emergency Use Authorizations for Medical Devices; 2024. https://www.fda.gov/medical-devices/emergency-use-authorizations-medical-devices/monkeypox-mpox-emergency-use-authorizations-medical-devices. Accessed on 15 January 2025.

[18] FIND. Outbreaks tests directory; 2024. https://www.finddx.org/tools-and-resources/dxconnect/test-directory/. Accessed on 15 January 2025.

[19] Gavina K., Franco L.C., Khan H., Lavik J.-P., Relich R.F. (2023). Molecular point-of-care devices for the diagnosis of infectious diseases in resource-limited settings—a review of the current landscape, technical challenges, and clinical impact. J Clin Virol.

[20] Hardwick C.S.H. Clinical characterisation protocol for severe emerging infection: BioMed Central Ltd; 2020 [cited 2024]. 10.1186/ISRCTN66726260. Accessed on 15 January 2025.

[21] Byrne R.L., Aljayyoussi G., Greenland-Bews C., Kontogianni K., Wooding D., Williams C.T. (2023). Comparison of the analytical and clinical sensitivity of thirty-four rapid antigen tests with the most prevalent SARS-CoV-2 variants of concern during the COVID-19 pandemic in the UK. medRxiv.

[22] Platform C-NDRaE. Facilitating AcceLerated Clinical validation Of novel diagnostics for COVID-19 United Kingdom; 2024. https://emergeresearch.org/trial/falcon/. Accessed on 15 January 2025.

[23] Byrne R.L., Aljayyoussi G., Kontogianni K., Clerkin K., McIntyre M., Wardale J. (2022). Head-to head comparison of anterior nares and nasopharyngeal swabs for SARS-CoV-2 antigen detection in a community drive-through test centre in the UK. medRxiv.

[24] Thompson CR, Torres PM, Kontogianni K, Byrne RL, LSTM Diagnostic group, Noguera SV, Luna-Muschi A, Marchi AP, Andrade PS, dos Santos Barboza A, Nishikawara M. Multicenter Diagnostic Evaluation of OnSite COVID-19 Rapid Test (CTK Biotech) among Symptomatic Individuals in Brazil and the United Kingdom. Microbiology Spectrum. 2023 Jun 15;11(3):e05044-22. 10.1128/spectrum.05044-22 PMC10269675 37212699

[25] Centers for Disease Control and Prevention. Test Procedure: Monkeypox Virus Generic Real‐time PCR Test; 2022. https://stacks.cdc.gov/view/cdc/119661. Accessed on 15 January 2025.

[26] Michel J., Targosz A., Rinner T., Bourquain D., Brinkmann A., Sacks J.A. (2022). Evaluation of 11 commercially available PCR kits for the detection of monkeypox virus DNA, Berlin, July to September 2022. Eurosurveillance.

[27] Li Y., Zhao H., Wilkins K., Hughes C., Damon I.K. (2010). Real-time PCR assays for the specific detection of monkeypox virus West African and Congo Basin strain DNA. J Virol Methods.

[28] Li Y., Olson V.A., Laue T., Laker M.T., Damon I.K. (2006). Detection of monkeypox virus with real-time PCR assays. J Clin Virol.

[29] Pawar S.D., Kode S.S., Keng S.S., Tare D.S., Diop O.M., Abraham P. (2022). Replication of SARS-CoV-2 in cell lines used in public health surveillance programmes with special emphasis on biosafety. Indian J Med Res.

[30] Cepheid. Xpert® Mpox Datasheet — US EUA; 2022. https://www.cepheid.com/en-US/tests/tb-emerging-infectious-diseases/xpert-mpox.html. Accessed on 15 January 2025.

[31] Biosensor S. STANDARD M10 MPX/OPX. https://www.sdbiosensor.com/product/product_view?product_no=23008

[32] Biosensor S. STANDARD M10 SARS-CoV-2 instructions for use; 2023. https://www.sdbiosensor.com/product/product_view?product_no=122. Accessed on 15 January 2025.

[33] Parakatselaki M.-E., Alexi G., Zafiropoulos A., Sourvinos G. (2023). Evaluation of STANDARDTM M10 SARS-CoV-2 assay as a diagnostic tool for SARS-CoV-2 in nasopharyngeal or oropharyngeal swab samples. J Clin Virol.

[34] Ham S.Y., Jeong H., Jung J., Kim E.S., Park K.U., Kim H.B. (2022). Performance of STANDARD™ M10 SARS-CoV-2 assay for the diagnosis of COVID-19 from a Nasopharyngeal Swab. Infect Chemother.

[35] Cubas-Atienzar A.I., Kontogianni K., Edwards T., Wooding D., Buist K., Thompson C.R. (2021). Limit of detection in different matrices of 19 commercially available rapid antigen tests for the detection of SARS-CoV-2. Sci Rep.

[36] Innovation C. Xpert MPOX EUA package insert - instructions for use; 2023.

[37] Li D., Wilkins K., McCollum A.M., Osadebe L., Kabamba J., Nguete B. (2017). Evaluation of the GeneXpert for human monkeypox diagnosis. Am J Trop Med Hyg.

[38] Damhorst G.L., McLendon K., Morales E., Solis Z.M., Fitts E., Bowers H.B. (2024). Performance of the Xpert™ Mpox PCR assay with oropharyngeal, anorectal, and cutaneous lesion swab specimens. J Clin Virol.

[39] Pan Z.-Y., Wu Y.-J., Zeng Y.-X., Lin H., Xie T.-A., Li Y.-P. (2021). Pooled analysis of the accuracy of Xpert Ebola assay for diagnosing Ebola virus infection. BioMed Res Int.

[40] Evans C.A. (2011). GeneXpert—a game-changer for tuberculosis control?. PLoS Med.

[41] Rossney A.S., Herra C.M., Brennan G.I., Morgan P.M., O'Connell B. (2008). Evaluation of the Xpert methicillin-resistant Staphylococcus aureus (MRSA) assay using the GeneXpert real-time PCR platform for rapid detection of MRSA from screening specimens. J Clin Microbiol.

[42] Mancon A., Raccagni A.R., Gagliardi G., Moschese D., Rizzo A., Giacomelli A. (2024). Evaluation of analytical performance of the STANDARDTM M10 MPX/OPX assay for the simultaneous DNA detection and clade attribution of Monkeypox virus. Emerg Microbes Infect.

[43] Tarín-Vicente E.J., Alemany A., Agud-Dios M., Ubals M., Suñer C., Antón A. (2022). Clinical presentation and virological assessment of confirmed human monkeypox virus cases in Spain: a prospective observational cohort study. Lancet.

[44] Ouafi M., Regueme A., Alcaraz I., Riviere P., Bazus H., Salmon‐Rousseau A. (2023). Oropharyngeal samples versus lesion specimens at diagnosis in patients infected with monkeypox virus in Northern France. J Med Virol.

[45] Palich R., Burrel S., Monsel G., Nouchi A., Bleibtreu A., Seang S. (2023). Viral loads in clinical samples of men with monkeypox virus infection: a French case series. Lancet Infect Dis.

[46] Suñer C., Ubals M., Tarín-Vicente E.J., Mendoza A., Alemany A., Hernández-Rodríguez Á. (2023). Viral dynamics in patients with monkeypox infection: a prospective cohort study in Spain. Lancet Infect Dis.

[47] Organization W.H. (2023).

[48] Wu F., Oghuan J., Gitter A., Mena K.D., Brown E.L. (2023). Wide mismatches in the sequences of primers and probes for monkeypox virus diagnostic assays. J Med Virol.

